# Research of affordability to essential medicines for coronary heart disease in Ukraine

**DOI:** 10.1080/20523211.2025.2470841

**Published:** 2025-03-11

**Authors:** Natalia Bilousova, Natalia Tkachenko, Nataliia Kozhuharyova, Maryna Dolzhenko

**Affiliations:** a Pharmaceutical and Preventive Medicine Faculty, Department of Pharmacy, Shupyk National Healthcare University of Ukraine, Kyiv, Ukraine; b Faculty of Pharmacy, Department of Management and Economics of Pharmacy, Zhaporizhzhia State Medical and Pharmaceutical University, Zaporizhia, Ukraine; c Faculty of Medicine, Department of Cardiology, Shupyk National Healthcare University of Ukraine, Kyiv, Ukraine

**Keywords:** Pricing, cardiovascular diseases, pharmaceutical assistance, access to medicines, economic burden, burden on household budget

## Abstract

**Background:**

The issue of population access to medicines is relevant worldwide. Pandemics, natural disasters, wars negatively affect the population's access to medicines, as emphasised by the WHO and the UN.

**Methods:**

The analysis of scientific publications in the Ukrainian scientometric databases (NRAT, OUCI); Scopus, Web of Science, Pubmed, Medline, BMJ and Embase; the legal field for providing medical care to patients with CHD and comorbid conditions and its pharmaceutical component. The EML of Europe and Ukraine are compared; the clinical recommendations and pharmacotherapy of European/American Societies of Cardiology (ESC/AHA) and Ukrainian for patients with CHD and comorbid conditions are compared.

**Results:**

The prices of medicines that are not part of the ‘Affordable Medicines’ program and are included in the EML lists were analysed; their availability for Ukrainian patients in wartime conditions was determined. The legal field of providing medical care to patients with CHD and comorbid conditions has been formed. It was established that not all medicines specified in the EML are registered on the territory of Ukraine and included in the Program of Medical Guarantees (PMG).

**Conclusion:**

The PMG includes most of the EML medicines. The modern pharmacotherapy of CHD and comorbid conditions has a positive impact on the budget of the health care system in clinical practice proposed by the ESC/AHA was determined. The lists of medicines in the PMG in Ukraine, need to be revised on the basis of the Health Technology Assessment for further inclusion in the state program ‘Affordable Medicines' for long-term use by patients with CHD and comorbid conditions. These measures will improve the quality of pharmaceutical care for these patients.

## Background

Among the Sustainable Development Goals (SDGs), the Goal 3 focuses on reducing premature mortality from non-communicable diseases (NCDs) by 2030 through the prevention and treatment, including cardiovascular disease (CVD) and promoting mental health and well-being (Goal 3.4); access to safe, effective, high-quality and affordable essential medicines (Goal 3.8); supporting research and development of medicines from NCDs, providing access to affordable basic medicines for all citizens of any country (Goal 3B) (World Health Organization [WHO], n.d.b).

It should be noted that in foreign scientific sources, the concept of population access and availability to medicines is a multifaceted approach that defines a complex of actions aimed at determining:

– shares from the remainder of the pharmacy assortment in a certain pharmacological group of medicines, in particular in the case of CVD in a pharmacy or a health care facility (HCF);
– the share of the patient’s total income that is spent on pharmaceuticals;
– the proportion of patients who adhere to the prescribed pharmacotherapy (adherence to treatment);
– the time spent by the patient to the nearest pharmacy or health care center;
– parts of pharmaceuticals that do not meet quality requirements.

At the same time, it is noted that medicines are considered affordable if the burden on the household budget is no more than 20% (Wirtz et al., 2016, pp. 2076–2085). According to ‘affordability', the World Health Organization (WHO) defines ‘a certain amount of daily tariff rates of a low-skilled civil servant with the lowest salary', which is spent on one course of treatment for a certain disease (WHO, 2021).

So, if we take into account that medicines for CVD are prescribed to patients for life and according to certain standards of specialised medical care (MC), we understand that in order to achieve the goal of pharmacotherapy of drugs of such patients, subjected to adherence to treatment, the burden on the household budget can be significant.

Scientists from different countries define the ‘population access to basic medicines' as the ability to obtain the necessary medicines ‘physically and at an affordable price'. This approach may be limited by the lack of medicines in pharmacy and health centers, the lack of ability to buy or pay for medicines from one’s own budget (Yenet et al., 2023, pp. 443–458).

Among European countries in the world ranking among all the main causes of death from СHD according to the Global Health Ranking (World Life Expectancy, n.d.), Ukraine took the first place in 2021. Accordingly, the total number of deaths from coronary heart disease in Ukraine per 100,000 population was 49.82% of Ukrainian citizens under the age of 70. ‘Analysis of the situation in the field of public health' indicates the occurrence of financial problems of such patients, which prevents the population’s access to drugs, in particular in the case of cardiovascular diseases (OCHA, 2023). According to the results of the analysis of pharmacy sales during the period from March 2023 to March 2024, in monetary terms, there was a redistribution of drugs according to the ATC classification, and there was an increase in the sales volume in monetary terms of groups C09 ‘Agents acting on the renin-angiotensin system' by 19.7% and C01 ‘Cardiac medicines’ by 15.3%, respectively. However, the data of the company ‘Proxima Research' indicate a decrease in the consumption of pharmaceuticals in packages during this period (Kirsanov, 2024b). Obviously, the redistribution of sales volumes was influenced by the war in Ukraine, the socio-economic, mental state of the civilian population and veterans, the environmental consequences of the war and the adherence to the prescribed drug pharmacotherapy (Bilousova & Mykhalchuk, 2024, pp. 347–355).

The issue of public access to essential medicines is of great scientific interest. Thus, access to the main medicines through the national health care systems is considered in the scientific works of Swiss and American scientists Ozawa et al. (2019, pp. iii1–iii3).

American scientists V.J. Wirts, W.A. Kaplan and others study the impact of pharmacotherapy of medicines used in CVD on the household budget, in particular, the economic availability of medicines. They raise the issue of the lack of stability in health care systems in various countries of the world and better access of the population to medicines, raised by Belgian and Canadian scientists Chattu et al. (2023, pp. 40–46).

The issues of availability and price policy for the essential medicines in the public and private sectors of Asian countries are raised by Indonesian and Indian researchers Amin et al. (2024), Goyal & Gilhotra (2015, pp. 083–087).

This topic is no less interesting for Ukrainian scientists. Thus, the issue of providing the population of Ukraine with medicines for budget funds is revealed in the scientific works of domestic scientists Nizhenkovska et al. (2023, pp. 3–10). Monitoring of the economic availability of medicines used in CVD is described in scientific publications of Symonenko et al. (2021, pp. 79–86). The rationale for improving the population’s access to original medicines on the basis of Health Technology Assessment (HTA) is revealed in the scientific studies of Ukrainian scientists Piniazhko et al. (2020, pp. 45–58). However, no systematic studies have been conducted to determine the total cost of drug pharmacotherapy in patients with ischemic heart disease on the household budget in Ukraine.

Therefore, the issue of studying the access of Ukrainian citizens with СHD to medicines in terms of the impact on the household budget, in particular ones with СHD, used for these pathological conditions in accordance with the unified clinical protocol of primary, secondary (specialised) and tertiary (highly specialised) medical care ‘Stable coronary heart disease' during the war in Ukraine.

This study was conducted by researchers in Ukraine during the ongoing war with Russian invaders, which began on February 24, 2022. Throughout the study, a consensus was reached among all participants. Common challenges were identified that hinder the provision of high-quality medical and pharmaceutical care and affect treatment adherence among patients with CHD with comorbid conditions.

Practical experience indicates that Ukrainian patients tend to adhere to treatment regimens for a limited period following the initiation of pharmacotherapy for CHD. However, upon experiencing an improvement in their well-being, patients with CHD and comorbid conditions often discontinue parts of their prescribed pharmacotherapy. One probable reason for this low adherence could be the significant financial burden on patients’ budgets, exacerbated by the reduced socio-economic conditions caused by the war in Ukraine (Bilousova & Dolzhenko, 2024, pp. 6–8). These observations served as the impetus for conducting this study.

## Methods

Scientific publications placed in Ukrainian information and scientific databases (NRAT, OUCI) and scientometric databases Scopus, Web of Science, PubMed, MedLine, BMJ and Embase served as the information basis for achieving the research goal. The research was conducted in several stages. To search for scientific data, we employed the narrative review method, focusing on peer-reviewed scientific sources with well-described methodologies in Ukrainian and English. A simple search was conducted using the following keywords: ‘pricing,' ‘cardiovascular diseases,' ‘coronary heart disease,' ‘pharmaceutical assistance,' ‘access to medicines,’ ‘economic burden,' and ‘burden on household budget,' covering publications from the past 10 years. The exclusion criteria included scientific publications that were indirectly related to the selected research topic and duplicate publications.

**The first stage** is the analysis of regulatory and legal documents of Ukraine to establish the legal framework for MC for patients with CHD and comorbid conditions and its pharmaceutical component. To achieve this, we conducted a search using the following keywords: ‘reimbursement' (13 documents), ‘medical guarantee program’ (59 documents), ‘state price regulation' (11 documents), ‘cardiovascular diseases’ (4 documents), ‘essential medicines' (28 documents), and ‘standards of medical care.' The search was performed on the websites of the Verkhovna Rada of Ukraine in the ‘Legislation' section, subsection ‘Regulatory and legal framework of Ukraine’ (Parliament of Ukraine, n.d.), and the State Expert Center of the Ministry of Health of Ukraine in the ‘Standardization in the field of health care' section, subsection ‘Standardization of medical care,' within the register of ‘Medical and Technological Documents' (State Expert Center of the Ministry of Health of Ukraine, n.d.). The topic of the search was ‘Coronary heart disease' (3 documents).

A total of 118 regulatory and legal documents were identified. The exclusion criteria included documents that were repeated with different dates of amendments. Only regulatory and legal documents with the most recent update date at the time of writing the article were used. As a result, 13 regulatory documents were selected for further analysis.

**The second stage** is a comparative analysis of the lists of the main medicines of European countries and Ukraine, as well as European, American and Ukrainian clinical recommendations for patients with CHD and comorbid conditions. For the comparative analysis of essential medicines lists in European countries and Ukraine, we utilised data from the World Health Organization (WHO) (Global Essential Medicines, n.d.; WHO, n.d.a) and the Ukrainian National List of Essential Medicines, including its amendments (Cabinet of Ministers of Ukraine, 2021a), as available at the time of the study.

For the comparative analysis of European, American, and Ukrainian clinical recommendations for patients with coronary heart disease and comorbid conditions, we used the most recently updated clinical guidelines published on the websites of the European Society of Cardiology (Byrne et al., 2023; Marx et al., 2023, pp. 4043–4140), the American Heart Association (Virani et al., 2023), and the Unified Clinical Protocol for Primary, Secondary (Specialised), and Tertiary (Highly Specialised) Medical Care, titled ‘Stable Coronary Heart Disease' (Ministry of Health of Ukraine, 2021a) From these sources, we identified the pharmaceutical component of the recommendations.

Subsequently, we compared the list of medicines included in the European, American, and Ukrainian clinical recommendations. To exclude medicines not registered in Ukraine, data from the State Register of Medicinal Products Registered in Ukraine were referenced (State Register of Medicinal Products of Ukraine, n.d.). The exclusion criteria included medicinal products that were not registered in Ukraine (State Register of Medicinal Products of Ukraine, n.d.).

**The third stage** is the analysis of pharmacotherapy for patients with coronary artery disease and comorbid conditions in Ukraine and in European countries to determine compliance with EML lists. We compared the list of medicines registered in Ukraine, identified during the second stage, with the National List of Essential Medicines (Cabinet of Ministers of Ukraine, 2021b, 2024a) and the Essential Medicines Lists (EML) of European countries (WHO, n.d.a, 2023) The exclusion criteria included medicines that were not registered in Ukraine (State Register of Medicinal Products of Ukraine, n.d.).

**The fourth stage** is the analysis of the prices of drugs that are not included in the ‘Affordable Medicines' program, are part of the EML lists and their availability for Ukrainian patients in wartime conditions. To achieve this, we divided the list of medicines used for the treatment of coronary heart disease (CHD) into two categories: essential medicines (Cabinet of Ministers of Ukraine, 2021a, 2024b) and a list of medicines covered by the state ‘Affordable Medicines' program (Cabinet of Ministers of Ukraine, 2023a). In the next step, we excluded medicines from the essential medicines list (Cabinet of Ministers of Ukraine, 2021b, 2024b) that were included in the ‘Affordable Medicines’ program (Cabinet of Ministers of Ukraine, 2023b).

We then evaluated the price variability (price stability) of medicines available on the Ukrainian pharmaceutical market that were not included in the ‘Affordable Medicines' program (Cabinet of Ministers of Ukraine, 2023b). For this analysis, we used the minimum and maximum prices available at the time of the study on a popular Ukrainian online platform for ordering medicines, which enables patients to purchase medicines at lower prices than those offered in physical pharmacies, regardless of their location in the country (Online platform for finding medicines in Ukraine, 2024). The liquidity ratio was calculated using the following formula:

$$\mathrm{RI}=\frac{P_{\max}-P_{\min}}{P_{\min}}\;\mathrm{where}$$

Rl – the liquidity ratio of medicines; *P*_max_ – the maximum medicines price; *P*_min_ – the minimum medicines price.

This liquidity ratio was used to analyze the availability of medicines for different categories of the population based on the minimum subsistence level and minimum wage. To determine the income levels of the population, we referred to official state websites of the Ministry of Finance of Ukraine (Ministry of Finance of Ukraine, 2024a). Two categories of the Ukrainian population were identified: those living on the subsistence minimum and those earning the minimum wage, as established by Ukrainian legislation (Ministry of Finance of Ukraine, 2024b). The accumulation, adjustment, systematization, and visualisation of the results were conducted using Microsoft Office Excel spreadsheets.

The following methods were employed: synthesis, systematization, generalisation, deduction, induction, comparative analysis, information-analytical methods, content analysis, as well as price and pharmacoeconomic analysis.

## Results

### Analysis of regulatory and legal documents of Ukraine

We have previously formed the legal field of MC for Ukrainian patients with CHD (Bilousova & Mykhalchuk, 2024, pp. 347–355) with the pharmaceutical component being singled out. It has been established that since the beginning of Russian aggression on the territory of Ukraine (since 2022), there have been constant legislative changes taking into account the conduct of military operations in occupied frontline territories and the implementation of European norms and requirements for Ukrainian medical practice.

In the pre-war period, Ukraine adopted the ‘State Strategy for the Implementation of the State Policy of Providing the Population with Medicines for the Period until 2025' was adopted, which takes into account the ways to overcome the identified problems of the population’s access to medicines through state price regulation, the cost reimbursement system under state guarantees of public medical care (MGP) and reimbursement (Program ‘Affordable Medicines'), medicines supply systems and ensuring the quality and rational use of medicines. Regular and systematic updating of clinical recommendations and protocols for the provision of medicines ensure the availability of ‘original (innovative)' medicines for the population (Cabinet of Ministers of Ukraine, 2018).

Our analysis of current Ukrainian legislation in the field of pharmaceutical care revealed that, at present, several drug lists are used in Ukraine for the treatment of patients with cardiovascular diseases (CVD). These lists regulate the population's access to medicines and ensure the availability of drugs in the assortment of healthcare institutions and pharmacies:

- National list of the essential medicines, which is updated every six months (Cabinet of Ministers of Ukraine, 2023b, 2024a);
– Nomenclature of pharmaceuticals and medical devices according to the direction ‘Medicines and medical devices for health care institutions to ensure the treatment of patients with cardiovascular and cerebrovascular diseases' (Ministry of Health of Ukraine, 2021a; Cabinet of Ministers of Ukraine, 2024b);
– List of medicines and medical devices offered for reimbursement under the program of state guarantees of medical care for the population (Cabinet of Ministers of Ukraine, 2021a, 2022).

In addition, patients with conditions after prosthetic surgery of heart valves and after myocardial infarction during the first six months in cases of outpatient treatment are provided with free prescription medicines (Cabinet of Ministers of Ukraine, 2023b).

It should be noted that according to the data of the World Bank, after the start of the war in Ukraine, as of December 2023, 63% of citizens experienced financial difficulties, and 25% did not have sufficient funds to provide food for households and, accordingly, access to health care (OCHA, 2024; The World Bank, 2024). This situation directly affects the solvency of Ukrainian patients to buy medicines used for CVD at their own expense.

**Figure 1.**
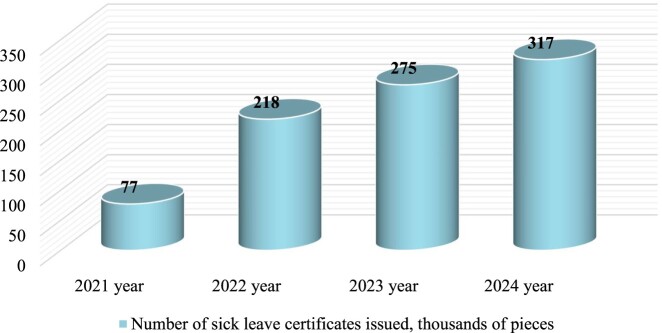
Dynamics of issued medical certificates of temporary disability in the category ‘increase in the number of signed disability certificates' by specialty cardiologist, interventionalist in the subcategory ‘general disease/injury' (Statistics of the creation of medical reports on temporary disability, n.d.)

Additionally, given that statistical data on the incidence and mortality rates of the population due to cardiovascular diseases during the war in Ukraine are not publicly available, we relied on electronic medical data accessible for public use and stored in the cloud environment of the National Health Service of Ukraine (Figure 1) (Statistics of the creation of medical reports on temporary disability, n.d.). The analysis revealed that, at the time of the study, there was a positive trend in the issuance of medical conclusions on temporary disability, specifically in the category ‘increase in the number of signed disability certificates' under the specialty of cardiologist and interventionalist within the subcategory ‘general disease/injury.’

Thus, based on the results of the analysis of current regulatory and legal documents, a number of legislative acts have been identified that guarantee the policy of providing the population with medicines for the period until 2025 (Cabinet of Ministers of Ukraine, 2018). However, the number of cardiovascular events in Ukraine has reportedly increased more than fivefold during the war. Hypothetically, one potential reason for this, as highlighted in WHO documents (OCHA, 2024; WHO, 2021, 2023), could be the limited access of the population to medicines. Given that the WHO methodology for assessing access to medicines cannot be fully implemented during wartime in Ukraine, we concentrated our efforts on applying deductive and inductive research methods.

### Comparative analysis of the lists of basic drugs of European countries and Ukraine

Given that European integration processes are underway in Ukraine, we compared the lists of essential medicines of European countries (EML) with the list of essential medicines in Ukraine. According to the results of a comparative analysis of the lists of the essential medicines of European countries and Ukraine, a significant difference was established. Thus, the largest number in the list of essential medicines is observed in Portugal (905), and the smallest – in Ukraine (278). Portugal also leads in terms of the number of differences in the Global List of Essential Medicines (EML) (807), and Ukraine has the greatest overlap in the list of essential medicines. It is worth noting that the compatibility of the lists of the essential medicines is also minimal in Portugal (28%) and maximal in Ukraine (80%). No less interesting are the data on health care expenses per capita. The minimum costs are observed in Ukraine – 584 US dollars and the maximum – in Sweden (5219 US dollars). It should be noted that the current Ukrainian legislation defines a list of reference countries. Accordingly, the maximum permissible prices for the dispensing of medicines for reimbursement in Ukraine are calculated based on the maximum permissible prices of these countries (Cabinet of Ministers of Ukraine, 2021a). The results of a comparative analysis of the lists of the essential medicines of reference countries (the Republic of Poland, the Slovak Republic, the Czech Republic, the Republic of Latvia, etc.) also testify to discrepancies with the number, differences and % coincidence with the EML, and the per capita costs in these countries amount to 1,580 US dollars (Table 1) (Cabinet of Ministers of Ukraine, 2021a; Global Essential Medicines, n.d.; WHO, n.d.a).

**Table 1.** Comparative characteristics of the essential medicines included in the EML in European countries and in Ukraine (Cabinet of Ministers of Ukraine, 2021b; Global Essential Medicines, n.d.; WHO, n.d.b, 2023).

Country	Quantity of medicines in the mandatory list of pharmaceuticals	Quantity of differences from the global list of pharmaceuticals	Compatibility, %	Healthcare costs per capita, $
Ukraine	278	242	80	584
Republic of Bulgaria	361	547	31	1399
Albania	214	386	56	615
Lithuania	339	447	45	1718
Slovenia	441	501	40	1570
Republic of Romania	635	587	36	1079
Montenegro	452	342	57	888
Republic of Latvia	304	464	41	940
Bosnia/Herzegovina	304	464	41	940
Macedonia	390	368	55	851
Republic of Poland	441	501	40	1570
Czech Republic	441	501	40	1570
Serbia	441	501	40	1570
Croatia	441	501	40	1570
Slovak Republic	441	501	40	1570
Estonia	405	507	38	1668
Malta	607	531	40	3072
Sweden	289	417	49	5219
Portugal	905	807	28	2690

So, taking into account the above, we observe that in Ukraine, per capita healthcare spending allocated by the public sector is almost three times lower than in reference countries. And the number of differences from the Global List of Medicines is half as low as in reference countries, which indicates a lack of flexibility in making political decisions regarding state regulation of providing the population with medicines.

### Analysis of pharmacotherapy of patients with coronary artery disease with comorbid conditions in Ukraine and European countries to determine compliance with EML lists

It should be noted that the standards of medical care for patients with CHD in Ukraine are developed in accordance with the clinical recommendations of the European Society of Cardiology and are revised every 5 years, which indicates an insufficiently rapid response to changes in evidence-based data with the rapid development of modern medical practice. The last update of the unified clinical protocol ‘Coronary Heart Disease' in Ukraine took place in 2021 (Ministry of Health of Ukraine, 2021b), therefore, general practitioners and family medicine physicians take into account the latest recommendations of the European Society of Cardiology and the American Heart Association (AHA). We have highlighted the pharmaceutical component in the standards of medical care for patients with CHD with comorbid conditions.

An analysis of European and American clinical recommendations for patients with coronary heart disease shows that the pharmacotherapy of medicines is being constantly updated and involves the simultaneous use of five to ten or more medicines (EURObservational Research Program EUROASPIRE V) (Kotseva, 2020a). Thus, in 2023, the European Society of Cardiology (ESC) proposed clinical recommendations for the management of patients with acute coronary syndrome (Byrne et al., 2023); CHD and diabetes and comorbid conditions (Marx et al., 2023), in which the following groups of medicines according to the ATC classification (Anatomical Therapeutic Chemical) are proposed before providing pharmaceuticals: C01 – C03, C07 – C10, B01, A10 (agents affecting the cardiovascular system; antithrombotic medicines; sodium-glucose co-transporter inhibitors 2). In Ukraine, pharmacotherapy of patients with coronary heart disease and comorbid conditions is carried out in accordance with clinical recommendations, guidelines and protocols for providing medical care to the relevant groups of patients (Ministry of Health of Ukraine, 2021b).

Our analysis of pharmacotherapy in Ukraine shows that with an acute shortage of budget funds and the minimum spending on health care per capita among European countries, the list of EML in Ukraine coincides by 80% with the Global List of EML. However, modern approaches to pharmacotherapy in accordance with Ukrainian standards of medical care for patients with CHD and ESC/AHA clinical recommendations require improvement of processes in the pharmaceutical supply of medicines and updating of EML lists to further provide better access of the country’s population with CVD to medicines (Bilousova & Mykhalchuk, 2024, pp. 347–355).

Based on the results of our analysis of drug therapy for primary and secondary MC for patients with CHD and comorbid conditions, we found that the EML list includes 28 medicines and fixed combinations in tablet form in various dosages and are used to prevent polypharmacy and increase adherence to treatment (Byrne et al., 2023; Marx et al., 2023, pp. 4043–4140) in of the specified group of patients (lisinopril/amlodipine, lisinopril/hydrochlorothiazide (HCTZ), telmisartan/amlodipine, telmisartan/HCTZ, acetylsalicylic acid (ASC)/atorvastatin/ramipril, ASC/simvastatin/ramipril/atenolol/HCTZ, atorvastatin/amlodipine/perindopril). In Ukraine, the National List of Essential Medicines includes 32 medicines, together with fixed combinations in tablet form of lisinopril/HTCZ and telmisartan/amlodipine in various dosages, which are purchased by health centers at state funds for Ukrainian patients who are provided with secondary and tertiary MC for CHD. The remaining fixed combinations are not registered in Ukraine (State Register of Medicinal Products of Ukraine, n.d.) and are not included in the reimbursement list (Cabinet of Ministers of Ukraine, 2021a, 2022).

In addition, 16 medicines (Table 2) are included to the list of medicines and medical devices that are subject to reimbursement under PMG (Table 2) (WHO, n.d.a; Cabinet of Ministers of Ukraine, 2021b, 2024a). For ease of comparison, we have distinguished the pharmaceutical component in the provision of primary and secondary medical care in medical standards and distributed medicines according to three lists of medicines. Empty cells in the table indicate the absence of a medicine in a specific list.

**Table 2.** Comparative analysis of pharmacotherapy for primary and secondary MC for patients with CHD and medicines that are sold under PMG reimbursement (Cabinet of Ministers of Ukraine, 2023a, 2024b; Ministry of Health of Ukraine, 2021b, 2023, 2024; WHO, 2023).

Drug therapy of primary MC with CHD	Drug therapy of secondary MC with CHD	Medicines, subjected to reimbursement	Medicines included into the National List of Pharmaceuticals	Global list of EML
**Nitroglycerin**				
Nitroglycerin	Nitroglycerin	Nitroglycerin	Nitroglycerin	Nitroglycerin
Isosorbide dinitrate	Isosorbide dinitrate		Isosorbide dinitrate	Isosorbide dinitrate
**Antiplatelet Agents**				
ASC	ASC	ASC	ASC	ASC
Clopidogrel	Clopidogrel	Clopidogrel	Clopidogrel	Clopidogrel
	Prasugrel			
	Ticagrelor			
**Beta-blockers**				
Esmolol	Esmolol	Bisoprolol	Bisoprolol	Bisoprolol
Metoprolol	Metoprolol	Metoprolol	Metoprolol	Metoprolol
			Atenolol	Atenolol
Propranolol	Propranolol			Propranolol
		Carvedilol	Carvedilol	Carvedilol
**Calcium Channel Blockers**				
Amlodipine	Amlodipine	Amlodipine	Amlodipine	Amlodipine
Verapamil	Verapamil	Verapamil	Verapamil	Verapamil
Diltiazem	Diltiazem		Diltiazem	
Nifedipine	Nifedipine	Nifedipine	Nifedipine	
**Anticoagulants**				
Apixaban	Apixaban		Apixaban	Apixaban
Warfarin	Warfarin	Warfarin	Warfarin	Warfarin
Heparin	Heparin		Heparin	Heparin
Dabigatran	Dabigatran		Dabigatran	Dabigatran
Enoxaparin	Enoxaparin		Enoxaparin	Enoxaparin
Rivaroxaban	Rivaroxaban		Rivaroxaban	Rivaroxaban
Phenindione	Phenindione		Phenindione	Phenindione
Fondaparinux	Fondaparinux		Fondaparinux	Fondaparinux
**Lipid-lowering Agents**				
Atorvastatin	Atorvastatin	Simvastatin	Simvastatin	Simvastatin
Ezetimibe	Ezetimibe			Atorvastatin
Rosuvastatin	Rosuvastatin			Pravastatin
**Angiotensin II Receptor Blockers**				
Valsartan	Valsartan	Losartan	Losartan	Losartan
**ACE Inhibitors**				
Captopril	Captopril	Enalapril	Captopril	
Lisinopril	Lisinopril		Enalapril	Enalapril
Ramipril	Ramipril			
**Other Cardiological Agents**				
Ivabradine	Ivabradine			
Ranolazine	Ranolazine			
Sildenafil	Sildenafil		Sildenafil	
**Diuretics**				
		HCTZ	HCTZ	HCTZ
		Furosemide	Furosemide	Furosemide
			Torasemide	Indapamide
			Indapamide	
**Mineralocorticoid Receptor Antagonists**				
		Spironolactone	Spironolactone	Spironolactone

We found out that the electronic Global List of EML contains 45 medicines that are used in CVD. 37 medicines (82.22%) from 45 drugs proposed by EML and from 45 drugs proposed by EML are registered from the data on pharmacotherapy in Ukraine, 16 medicines (35.56%) of the registered ones are released to the population of the country under reimbursement. 32 medicines (71.11%) out of 45 drugs recommended by EML, were included in the National list of basic medicines. Thus, 21 medicines (46.67%) that are prescribed for long-term permanent admission for CVD are purchased by Ukrainians at their own expense.

**Figure 2.**
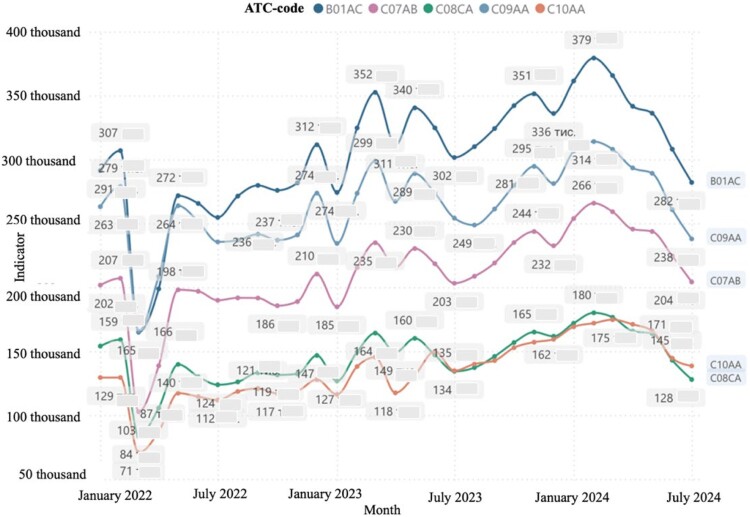
The dynamics of the quantity of sold packages of pharmaceuticals according to ATC classification under the ‘Affordable Medicines' program (National Health Service of Ukraine, 2024).

**Figure 3.**
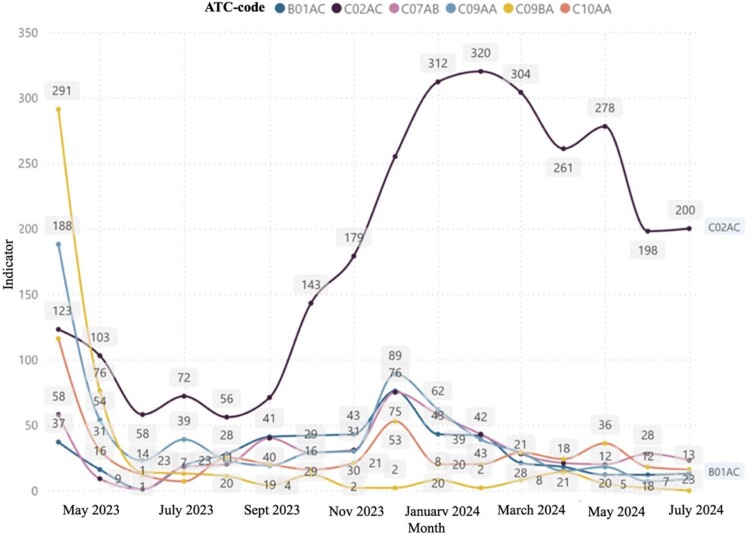
The dynamics of the number of sold packages of pharmaceuticals according to ATC classification at the expense of individuals (National Health Service of Ukraine, 2024).

Our own observations indicate that during the war in Ukraine, the able-bodied part of the population and able-bodied people of retirement age work an eight-hour workday without rest or holidays. Ukrainians do not always have time to visit doctors to write prescriptions and receive primary medical care. As a result, they frequently lack time to visit doctors for prescriptions and primary healthcare. Consequently, a portion of the Ukrainian population purchases medicines for cardiovascular diseases without prescriptions and does not participate in the ‘Affordable Medicines' program, which is not reflected in Figures 2 and 3.

Thus, we observe that of the 31 drugs proposed for the pharmacotherapy of CHD at the primary and secondary levels of medical care, less than half are eligible for reimbursement. The data we obtained indicate that nearly 50% of CHD pharmacotherapy is not covered by the ‘Affordable Medicines’ program, which negatively impacts household budgets, particularly for socially vulnerable segments of the population, including internally displaced persons from temporarily occupied territories. As a result, adherence to treatment among such patients deteriorates.

Simvastatin, enalapril, and valsartan, which are included in the ‘Affordable Medicines’ program (Cabinet of Ministers of Ukraine, 2021b, 2022) and are also proposed in the Global EML List (WHO, 2023), remain controversial. As an alternative to simvastatin, the Global EML List (WHO, 2023) recommends considering atorvastatin and pravastatin, while rosuvastatin is recommended for use in fixed combinations. Additionally, we note that direct oral anticoagulants, sodium-glucose cotransporter 2 inhibitors, and finerenone are not eligible for reimbursement under the Affordable Medicines program (Cabinet of Ministers of Ukraine, 2021b, 2022).

According to the data of the National Health Service of Ukraine (NHSU) (National Health Service of Ukraine, 2024) for the period from January 2022, after the beginning of the war in Ukraine, there is a positive trend in the dispensing of medicines under reimbursement (the ‘Affordable Medicines' Program) according to ATC groups B01АС, С07АВ, С08СА, C10AA (Figure 1).

### Analysis of prices for drugs that are not included in the ‘Affordable Medicines' program, are included in the EML lists, and their availability for Ukrainian patients during wartime

However, a significant amount of medicines (46.67%) used for the treatment of CVD is purchased at the expense of individuals, which causes an additional burden on the budget of Ukrainian households. There is a general redistribution of medicines applied for CHD with comorbid conditions towards the ‘Affordable Medicines' (Cabinet of Ministers of Ukraine, 2021b, 2022). Program from January 2023, however the C02AC group (imidazoline receptor agonists) is singled out. This fact indicates the positive dynamics of sales growth in packages. It should be noted that imidazoline receptor agonists are used as additional pharmacotherapy in hypertension to achieve target blood pressure levels, which subsequently reduces the risk of progression of hypertension and the development of CHD and related conditions (Figure 2) (National Health Service of Ukraine, 2024). In addition, we note that in Figure 2 displayed medicines that are not part of the ‘Affordable Medicines' program. Fixed medicines combinations, which are now widely used in the specified group of patients in order to increase adherence to treatment, are also not taken into account.

As a side note, the minimum monthly wage in Ukraine as of 1 April 2024 is UAH 8,000 according to the Ministry of Finance of Ukraine, which equivalents $195.1 (Ministry of Finance of Ukraine, 2024a). The minimum subsistence for socially vulnerable segments of the population as of 1 April 2024 is UAH 2,920, which is $71.2 (Ministry of Finance of Ukraine, 2024b). The official exchange rate of the hryvnia to the US dollar according to the data of the National Bank of Ukraine as of 1 August 2024 is 41.0063 (National Bank of Ukraine, 2024). Therefore, the average monthly burden on the household budget of pharmacotherapy of medicines used for CHD and comorbid conditions should be no more than UAH 1,600 (US$ 39) for working citizens and UAH 584 (US$ 14.24) for socially vulnerable segments of the population (pensioners, people with disabilities, internally displaced persons from temporarily occupied and frontline territories, etc.) to be accessible to Ukrainian patients.

The analysis of the prices for medicines that are not part of the ‘Affordable Medicines' program and are used in primary and secondary MC for patients with CHD and comorbid conditions, according to the Global EML list, shows that all medicines are not liquid in price (Table 3).

**Table 3.** Analysis of the prices of pharmaceuticals that are not included into the ‘Affordable Medicines' program and are used in primary and secondary MC for patients with IHD and comorbid conditions (Online platform for finding medicines in Ukraine, 2024).

ATC Classification Group	Medicinal Product, Dosage	Minimum Price, UAH	Maximum Price, UAH	Average Price, UAH	Liquidity Ratio
C10B X06	ASC/atorvastatin/ramipril tablets 100/20/2.5 mg	501,1	714,4	607,75	0,43
C10B X06	ASC/atorvastatin/ramipril tablets 100/20/5 mg	573,59	880,65	727,12	0,54
C10B X06	ASC/atorvastatin/ramipril tablets 100/20/10 mg	551,99	765,91	658,95	0,39
С09В Х01	Perindopril/indapamide/ amlodipine 5/1.25/5 mg	133,91	396,52	265,22	1,96
С09В Х01	Perindopril/indapamide/ amlodipine 10/2.5/10 mg	190,88	474,21	332,55	1,48
С09В Х01	Perindopril/indapamide/ amlodipine 10/2.5/5 mg	183,91	446,49	315,2	1,43
C10A A05	Atorvastatin 10 mg	65,55	215,12	140,34	2,28
C10A A05	Atorvastatin 20 mg	91,71	261,6	176,66	1,85
C03B A04	Chlortalidone 25 mg	100,6	188,13	144,37	0,87
C03B A11	Indapamide 1.5 mg	91	230,46	160,73	1,53
C03B A11	Indapamide 2.5 mg	53	220,4	136,7	3,16
C09B B03	Lisinopril/amlodipine 10/5 mg	124,26	254,89	189,58	1,05
C09B B03	Lisinopril/amlodipine 20/5 mg	168,95	455,01	311,98	1,69
C09B B03	Lisinopril/amlodipine 20/10 mg	218,34	665,95	442,15	2,05
С09В А03	Lisinopril/HCTZ 20/12.5 mg	89,23	159,39	124,31	0,79
С02А В01	Methyldopa 250 mg	193,25	334,62	263,94	0,73
C09D B04	Telmisartan/amlodipine tablets 40/5 mg	119,76	233,62	176,69	0,95
C09D B04	Telmisartan/amlodipine tablets 80/5 mg	170,29	284,4	227,35	0,67
C09D B04	Telmisartan/amlodipine tablets 80/10 mg	198,9	291,9	245,4	0,47
С0ЗС А04	Torasemide 5 mg	126,2	301,95	214,08	1,4
С0ЗС А04	Torasemide 10 mg	92,7	339,57	216,14	2,66
С0ЗС А04	Torasemide 20 mg	285,45	468,51	376,98	0,64
B01A F01	Rivaroxaban 2.5 mg	776,33	1208,07	992,2	0,56
B01A F01	Rivaroxaban 20 mg	775,84	1227,4	1001,62	0,58
B01A F01	Rivaroxaban 15 mg	823,72	1480,4	1151,5	0,8
B01A E07	Dabigatran etexilate 110 mg	517,65	752,2	634,93	0,45
B01A E07	Dabigatran etexilate 150 mg	548,86	817,94	683,4	0,49
B01 AF02	Apixaban 2.5 mg	935,12	1420,73	1177,93	0,52
B01 AF02	Apixaban 5 mg	542,32	1488,9	1015,61	1,75
C10B A06	Rosuvastatin/ezetimibe 10/10 mg	253,63	855	554,32	2,37
C10B A06	Rosuvastatin/ezetimibe 20/10 mg	339,13	1053,55	696,34	2,11
С01Е B17	Ivabradine 5 mg	147,89	488,78	318,34	2,31
С01Е B17	Ivabradine 7.5 mg	85,13	455,29	270,21	4,35
А10ВК01	Dapagliflozin 10 mg №30	921	1647,36	1284,18	0,79
A10B К03	Empagliflozin 10 mg №30	701,16	1168,5	934,83	0,67

It was determined that none of the studied medicines exhibit low (0 < Kl ≤ 0.1) or moderate (0.1 < Kl ≤ 0.3) price variability, which reflects the instability of the Ukrainian pharmaceutical market during the war. All medicines that are not eligible for reimbursement under the ‘Affordable Medicines' program (Cabinet of Ministers of Ukraine, 2021b, 2022) demonstrate high variability (Kl > 0.3), indicating disparities in access to medications and a lack of strict and transparent state of price regulation. This also points to the monopolisation of the pharmaceutical industry, including wholesale distribution, and excessive mark-ups on certain medicines. These issues are also highlighted in the strategic analysis of corruption risks in the process of conducting medical technology assessments in Ukraine (National Agency for Corruption Prevention, 2023).

## Discussion

The analysis of pharmacy sales based on the results of the first half of 2024 and the volume of retail sales shows that cardiac medicines (group C01) have increased in monetary terms by 10.9% (Kirsanov, 2024a). Among these medicines, the best-selling ones (TOP 20 medicines) in monetary terms were brand medicines with the International Nonproprietary Name (INN) rivaroxaban; valsartan and fixed combinations of perindopril/amlodipine/indapamide; perindopril/indapamide; captopril/HCTZ, which indicates the use in medical practice of medicines that are not included to the ‘Affordable Medicines' (Kirsanov, 2024b) program and are used by a sufficient segment of the population with CHD and comorbid conditions (Kirsanov, 2024b). Provided that if patients receive medicines under reimbursement and additionally buy fixed combinations of perindopril/amlodipine/indapamide (5th place in the TOP – 20 medicines), atorvastatin and rivaroxaban (2nd place in the TOP – 20 medicines), the burden on the budget of Ukrainian households will be 20% (UAH 1,600) for working strata of the population and 58% of the household budget for socially vulnerable segments of the population, which is 25% of Ukrainian citizens (OCHA, 2024). Therefore, the question of the availability of medicines for Ukrainian citizens in the conditions of war and socio-economic crisis is relevant at the moment and requires further improvement of state price regulation, and updating of the cost reimbursement system under the ‘Affordable Medicines' program.

We emphasize the strengths of our study. It was carried out on the basis of an integrated approach to the set research goal using various research methods, indicated in the materials and research methods section. The results of our study can be further used to improve state policy in the areas of strict state regulation of prices for medicines that are subject to reimbursement and included in the list of essential medicines, which adds strength to this study. Moreover, the research findings are backed by current Ukrainian regulatory documents. The comparison of Ukrainian practices with European and American standards adds a global perspective to our analysis. Additionally, the use of price calculations based on the liquidity coefficient enabled us to accurately assess price variability and the economic accessibility of medicines.

The data we gathered provide a clear understanding of the negative impact that the economic burden on the budgets of socially vulnerable population segments has on the full utilization of CHD pharmacotherapy and treatment adherence. This is consistent with the findings from the EUROASPIRE IV (Gyberg et al., 2015) and EUROASPIRE V (Kotseva et al., 2020b) studies, in which Ukrainian patients participated.

It should be noted that the pharmacoeconomic analysis of optimising secondary and tertiary cardiovascular prevention conducted in the EUROASPIRE III (De Smedt et al., 2012) and EUROASPIRE IV (De Smedt et al., 2018) studies demonstrated additional cost-effectiveness ratios (ICER) of €12,484 (EUROASPIRE III) and €52,968 (EUROASPIRE IV) per year of additional life adjusted for the quality of life (QALY) of patients. Therefore, improving the population's access to medicines, considering the economic burden on household budgets, can enhance adherence to treatment in patients with coronary heart disease (CHD) when effective pharmacotherapy is used. This, in turn, may reduce the number of cardiovascular events, hospitalisations, and the overall burden on the healthcare budget.

The authors acknowledge the methodological limitations of conducting a narrative review of scientific sources and did not aim to carry out a systematic review. Additionally, this study did not aim to investigate the physical accessibility of medicines. The results obtained were used to clarify the current economic burden on the budget of patients with CHD, with the intention of further investigating factors that affect adherence to treatment in these patients and advancing the scientific justification for a conceptual model of pharmaceutical care for such patients in Ukraine. This study is based on secondary data and does not include clinical or empirical data, which will be explored in our future research. We also emphasise the challenges in fully replicating the WHO methodology (WHO, 2021) for determining medicine accessibility under wartime conditions in Ukraine, which constitutes a limitation of our study.

## Conclusion

1. By means of a comparative analysis, it was found that not all medicines listed in the EML Global List of Medicines are registered on the territory of Ukraine and included in the PMG lists.
2. Based on the analysis of the pharmacotherapy of patients with ischemic heart disease and comorbid conditions, medicines that are not in the Global List of EML were identified. It has been established that the PMG includes the main part of medicines from the Global List of EML, but socially vulnerable segments of the Ukrainian population do not have financial (economic) access to the rest of medicines.
3. It was determined that the lists of medicines in Ukraine included in the PMG need to be revised on the basis of Health Technology Assessment for further inclusion into the state program ‘Affordable medicines' for a long-term use for patients with CHD and comorbid conditions. These measures will improve the quality of medical and pharmaceutical care, adherence for these patients.
4. The positive impact of the use of modern pharmacotherapy for CHD on the budget of the health care system in clinical practice proposed by the ESC, was established.
5. The positive impact of the use of modern drug pharmacotherapy for CHD of the budget of the health care system in clinical practice proposed by the ESC, was established. Confirmed by the data of the EUROASPIRE V observational study, in which Ukrainian patients participated, was established.

## Data Availability

All data generated and analyzed are included in this research article.
